# Cannabis Use and Neuroadaptation: A Call for Δ*^9^*-Tetrahydrocannabinol Challenge Studies

**DOI:** 10.3389/fpsyt.2022.870750

**Published:** 2022-04-14

**Authors:** Johannes G. Ramaekers, Eef L. Theunissen, Peter van Ruitenbeek, Natasha L. Mason

**Affiliations:** Department of Neuropsychology and Psychopharmacology, Faculty of Psychology and Neuroscience, Maastricht University, Maastricht, Netherlands

**Keywords:** cannabis, neuroadaptation, neurocognition, mesocorticolimbic circuit, cannabis abuse

## Abstract

Currently, the assessment of the neurobehavioral consequences of repeated cannabis use is restricted to studies in which brain function of chronic cannabis users is compared to that of non-cannabis using controls. The assumption of such studies is that changes in brain function of chronic users are caused by repeated and prolonged exposure to acute cannabis intoxication. However, differences in brain function between chronic cannabis users and non-users might also arise from confounding factors such as polydrug use, alcohol use, withdrawal, economic status, or lifestyle conditions. We propose a methodology that highlights the relevance of acute Δ^9^-tetrahydrocannabinol (THC) dosing studies for a direct assessment of neuroadaptations in chronic cannabis users. The approach includes quantification of neurochemical, receptor, and functional brain network changes in response to an acute cannabis challenge, as well as stratification of cannabis using groups ranging from occasional to cannabis-dependent individuals. The methodology allows for an evaluation of THC induced neuroadaptive and neurocognitive changes across cannabis use history, that can inform neurobiological models on reward driven, compulsive cannabis use.

## Introduction

Cannabis is the most commonly used illicit drug, with 4% of the global population reportedly using the substance (1). The prevalence of cannabis use is expected to increase following recent trends to legalize or decriminalize its use for recreational and therapeutic purposes (2, 3). Thus, as cannabis use increases and the perception of risk of use decreases (4), a pertinent question is how prolonged cannabis use affects the neurocognitive state, and whether there are long-term neurobiological consequences (5). Furthermore, as 10% of those who recreationally consume cannabis develop daily use patterns (6), it is prudent to understand neuroadaptations in the neuro-circuitry which may underlie this increase and persistence of use.

Neuroadaptation in chronic cannabis users has traditionally been evaluated with brain imaging measures in comparison to non-cannabis using controls. Many of these cross-sectional fMRI studies have revealed changes in functional connectivity (7–11), task-related brain activation (12–18) and neurotransmission (19–23) in various brain regions of chronic cannabis users, sometimes in association with cognitive deficits (24–29). However, findings have also been mixed as these studies suffer from the methodological problem that confounding factors (e.g., pre-existing differences, polydrug use, and differences in lifestyle) cannot adequately be controlled and therefore, in addition to cannabis use, might explain observed differences in brain function (30). Cross-sectional studies between chronic cannabis users and non-cannabis users therefore cannot be taken as the sole approach for studying the effects of repeated cannabis use on brain function and its associated neurocognitive state.

**FIGURE 1:**
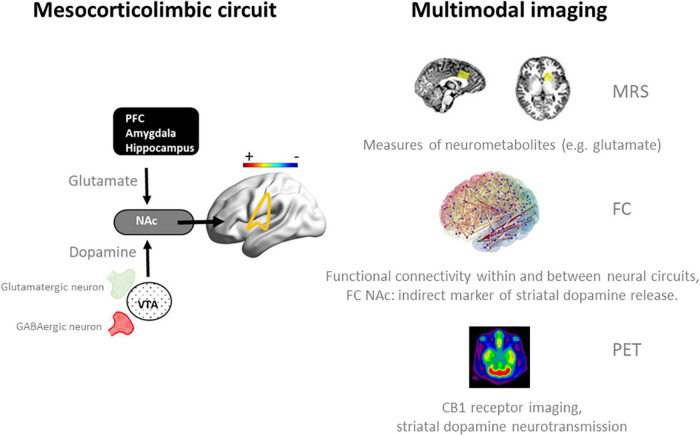
Schematic representation of neurotransmission and functional connectivity in the mesocorticolimbic circuit in the normal brain (32) (left panel) and multimodal imaging measures (right panel) that can be employed to assess dynamics within this circuit during an acute challenge with THC and when sober. Magnetic Resonance Spectroscopy (MRS) has been successfully used to quantify frontal and striatal glutamate concentrations in cannabis users during intoxication and when sober (33, 34). fMRI measures of functional connectivity (FC) have provided functional associations within and between neural networks in cannabis users during intoxication (33, 35, 42) and when sober (15–17, 42), and can be used as an indirect marker of striatal dopamine release during cannabis intoxication (33, 81). Positron emission tomography (PET) can be used to determine CB1 receptor density at glutamatergic and GABAergic neurons in the striatum of cannabis users (60, 62) and to determine dopamine displacement at D2/D3 receptors as a measure of dopamine transmission during cannabis intoxication (36). NAc, nucleus accumbens; PFC, prefrontal cortex.

**FIGURE 2:**
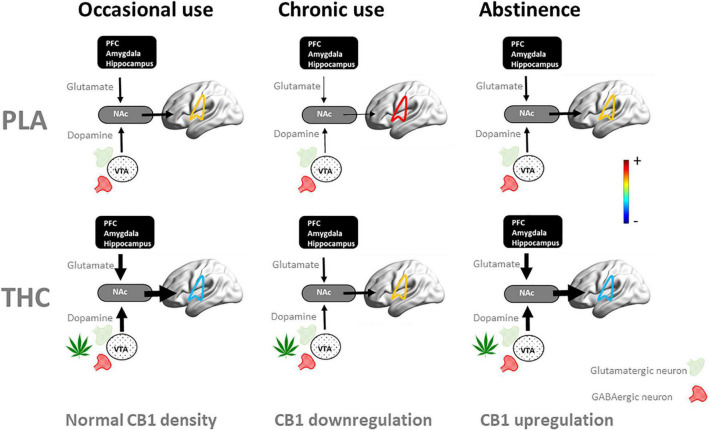
Schematic representation of the dynamics of the neuroadaptive state of the mesocorticolimbic circuit when challenged with placebo (PLA) and THC as users proceed from occasional to chronic cannabis use and into abstinence. In occasional users, an acute THC challenge increases striatal levels of dopamine and glutamate (33–35) through stimulation of CB1 receptors at presynaptic GABAergic and glutamatergic receptors, leading to hypoconnectivity of the mesocorticolimbic circuit (here shown superimposed on brain) and neurocognitive dysfunction (33, 35). With repeated cannabis use, CB1 receptors are downregulated as a neuroadaptive response to CB1 receptor overstimulation (60, 62). In chronic users, this leads to reduced striatal dopamine (40, 41) and glutamate (19–22), as well as hyperconnectivity within the mesocorticolimbic circuit (9, 42, 82) and neurocognitive dysfunction when sober (13, 24, 27, 28). In these users, stimulation of CB1 receptors normalizes striatal dopamine and glutamate concentrations, functional connectivity and neurocognitive function during acute THC intoxication (35). With prolonged abstinence of cannabis, CB1 receptors upregulate (60, 62) and potentially normalize the neuroadaptive and neurocognitive state (63, 64, 66). NAc, nucleus accumbens; PFC, prefrontal cortex.

Alternatively, we propose that neuroadaptive changes in cannabis users can more selectively be studied in response to an acute challenge with Δ^9^ -tetrahydrocannabinol (THC). Ideally, such studies would follow a multimodal imaging approach that includes resting state fMRI to assess functional connectivity, PET imaging to profile CB1 receptor densities, and magnetic resonance spectroscopy to quantify neurometabolites in neural circuits in which neuroadaptions are prominent, such as the mesocorticolimbic circuit (31) (Figure 1). Neural changes to an acute THC challenge should be assessed as a function of cannabis use frequency as there is a notion that neural mechanisms underlying acute and long-term cognitive deficits are interrelated and that the latter can be explained as a neuroadaptive response to the former (32). For example, an acute dose of THC has been found to increase glutamate (33–35) and dopamine (36–39) in the striatum of occasional users, whereas sober chronic cannabis users demonstrate a decrease of glutamate and glutamate-related metabolites (19–22), and lower levels of dopamine release (40, 41). Likewise, fMRI studies have repeatedly shown hypoactivation in the mesocorticolimbic circuit during acute THC intoxication (32, 33, 35) in occasional users but hyperactivation in sober, chronic cannabis users (12, 15–17, 26, 42). The intermediate mechanism might be CB1 receptor density that is known to fluctuate with cannabis frequency and represents a neuroadaptive response of the brain to regain homeostasis following sustained and repeated THC exposure (31). It can be hypothesized that this neuroadaptive response in chronic cannabis users is aimed to normalize cognitive function during THC intoxication, but causes underactivation of brain function when sober (32). The dynamics of this antipodal neural response underlying acute and long-term effects of cannabis can typically be studied in placebo-controlled THC studies as a function of cannabis use frequency as shown in Figure 2. This approach could be applied to a number of research issues that are closely associated with cannabis use disorder, as discussed below. These include assessments of neural mechanisms that underlie the progression to compulsive cannabis use, the development of tolerance, and their association to neurocognitive key-elements of addiction such as reward, craving, and cognitive control (43).

## Transition to Compulsive Cannabis Use

Acute THC studies can be employed to increase or confirm our understanding of the neural and psychological basis of the transition from initial cannabis use to compulsive use. Compulsive cannabis use occurs in parallel to the development of cannabis tolerance but their underlying neural mechanisms may differ. Preclinical studies have suggested that THC-induced dopamine release shifts from the ventral striatum to the dorsal striatum after repeated administration (44), a transition that has been associated with the development of dependence in humans (45). In addiction research, dorsal striatal activation dominance in drug use disorders has been associated with habitual, drug-seeking behavior (43, 46–49), while increased ventral striatal activation has been associated with increased responsiveness to drug-related cues and reward (50–52). Indeed, dependent and non-dependent cannabis users have shown increased responsiveness of the ventral striatum in response to cannabis-related cues (8, 45, 53–56) and increased striatal frontal coupling (45). Dependent users also exhibit increased dorsal striatal reactivity and decreased striatal limbic coupling during cannabis cue exposure (45). Shifts in ventral and dorsal activation as observed in cannabis users are thus intrinsically related to the frequency of acute cannabis intoxications. The development of the striatal response to cannabis cues should therefore also be studied and understood in the context of acute THC challenge studies in groups with varying cannabis use frequencies. Such studies could potentially monitor directly how acute THC intoxication affects ventral-dorsal striatal activation as a function of cannabis use history and further clarify the contributory roles of striatal dopaminergic and glutamatergic neurotransmission to excessive cannabis use and habit formation. The latter is of particular relevance, as a number of acute THC studies have shown that a single dose of THC attenuates the striatal response to rewards such as monetary incentives, cannabis marketing, and music (56–58) suggesting that THC-induced increments in striatal dopamine reduce salience and attentional processing of concurrent rewards (56). Acute THC challenge studies therefore would not only contribute to a fundamental understanding of ventral-dorsal striatal activation with increasing cannabis use, but also provide important pharmacological insights on how shifts in striatal balance can be prevented or controlled in order to avoid progression to compulsive cannabis use.

## Dynamics of Tolerance and Neurocognition

Neurocognitive impairments observed during cannabis intoxication are transient and dynamic over time, depending on cannabis use frequency (31). Understanding the dynamics of tolerance can be useful when trying to “tailor” the cannabis experience and develop dosing strategies which promote tolerance in patient populations where they do not want the “high” and neurocognitive impairment. Alternatively, dynamics of tolerance can also inform strategies to promote efficient “tolerance breaks” for individuals who want the “high” and the associated neurocognitive state, but wish to reduce the potential for addiction, as tolerance development promotes addictive behavior (31, 59).

The neurocognitive state of cannabis users is strongly associated with neuroadaptations in the mesocorticolimbic circuit that occur during acute intoxication, chronic use, and abstinence (see Figure 2). Chronic exposure to cannabis produces significant downregulation and desensitization of CB1 receptors in cannabis-dependent users relative to that in controls (60–62). This homeostatic response of the brain also potentially causes a state of underactivation and neurocognitive dysfunction in chronic cannabis users when sober (31, 63–65). Typically, such deficits rapidly decrease during abstinence and do not persist beyond 4–5 weeks (63, 66). It has been hypothesized that neurocognitive impairment in chronic users arise from a state of withdrawal during which CB1 receptors are downregulated and restrained from THC-related receptor stimulation (32). Subsequent CB1 receptor upregulation observed during withdrawal in chronic users (60) was indeed paralleled by an improvement in neurocognitive function (64). Interestingly, the only study to date that investigated the neuroadaptive response to an acute challenge in chronic cannabis users (35) suggested that stimulation of CB1 receptors subsequently normalizes striatal glutamate and dopamine transmission, functional mesocorticolimbic connectivity, and neurocognitive function (31, 32, 35). This neuroadaptive response may also explain the absence of neurocognitive impairment (i.e., tolerance) that is often reported in chronic cannabis users during THC intoxication (31, 67).

Neuroimaging studies can be instrumental in assessing the dynamics of CB1 receptor density and neurocognitive state before or after an acute THC challenge in groups of cannabis users who vary in their frequency of use. Such studies could establish downregulation of CB1 receptor density with increasing cannabis use and determine the impact of CB1 downregulation on mesocorticolimbic function and neurocognitive state, in the presence and absence of an acute THC challenge. Increased knowledge of how temporal changes in cannabis use frequency affect the dynamics of an individual’s response and neuroadaptations to an acute challenge with cannabis, will gain relevance with increasing recreational and medical use of cannabis. Frequency, dose, and duration of use to achieve or reverse tolerance are currently unknown but are important to define recreational use frequencies and medical dosing strategies at which the development of acute tolerance, persistence of neurocognitive deficits, and compulsive use can be avoided.

## Markers of the Neurocognitive State

Acute THC studies might also serve to identify neural markers of cognitive function that differentiate compulsive cannabis use from non-problematic cannabis exposure, or the impaired state from the non-impaired state. At present, there is no objective assessment that can classify the neurocognitive state in individual cannabis users. Recent studies have suggested, however, that acute THC intoxication as assessed by subjective ratings of “high” produces a reproducible signature change in brain function that can be detected with neuroimaging techniques (42, 68). The former study (68) conducted functional near-infrared spectroscopy (fNIRS) in cannabis users before and after receiving oral THC and placebo and found increased oxygenated hemoglobin concentration (HbO) in the prefrontal cortex of participants with a clinical rating of subjective intoxication. Machine learning models using fNIRS time course features and connectivity matrices identified the intoxicated state with 76.4% accuracy (68). The latter study (42) used a data-driven independent component methodology to analyze fMRI resting state data to extract a distinct spatial connectivity pattern of hypoconnectivity involving the dorsal attention, limbic, subcortical and cerebellum networks, and of hyperconnectivity between the default mode and ventral attention network, that was associated with the feeling of a subjective “high” during THC intoxication (42). That same study also revealed a broad state of hyperconnectivity within whole-brain networks in chronic cannabis users compared to occasional cannabis users, which might be reflective of an adaptive network reorganization following prolonged cannabis exposure. These acute THC studies suggest that neural fingerprints of cannabis intoxication and cannabis use history can be derived from neuroimaging data. Future studies with acute THC challenges might identify neurobiological features or phenotype characteristics of impaired and maladaptive behaviors that might arise from acute and chronic use of cannabis. Such models might provide unique insights into emerging adaptations of distinct functional networks in users that progress from occasional to chronic cannabis use and underlie the development of a pathological state, such as cannabis use disorder.

## Concluding Remarks

While the effects of chronic cannabis exposure on brain function and cognition have become a focal point for research, much remains unknown about neuroadaptive responses to acute THC intoxication, and how these develop over time into chronic cannabis use. This paper posits that pharmaco-imaging studies should be considered to explore how neural responses to an acute THC challenge develop with repeated cannabis use and how such neuroadaptations relate to the development of cannabis tolerance, compulsive cannabis use, and their associated neurocognitive state. Such studies could also target additional factors that are known to moderate the neural response to an acute THC challenge, such a dose, potency, composition, and formulations of cannabis products as well the interaction with underlying pathological states in case of medical use (32). In principle, this approach could also be expanded on to assess neuroadaptations to other drugs of abuse. For example, acute and chronic alterations in neurotransmission and functional connectivity of the mesocorticolimbic circuit have been reported for cocaine (69–71), nicotine (72–76), and alcohol (77–80). The current proposal on how to assess and define neuroadaptations in cannabis users would also call for an international, multi-center research effort in order to include large samples of distinct cannabis user groups, ranging from novice and occasional users at the lowest end of the use frequency spectrum to daily, chronic users at the opposite extreme. It would offer a unique opportunity to develop an integrative, mechanistic view of long-term effects of cannabis on the brain as a neuroadaptive response to acute THC challenges or to the absence thereof.

## Author Contributions

All authors contributed to the manuscript and approved its content.

## Conflict of Interest

The authors declare that the research was conducted in the absence of any commercial or financial relationships that could be construed as a potential conflict of interest.

## Publisher’s Note

All claims expressed in this article are solely those of the authors and do not necessarily represent those of their affiliated organizations, or those of the publisher, the editors and the reviewers. Any product that may be evaluated in this article, or claim that may be made by its manufacturer, is not guaranteed or endorsed by the publisher.
